# Self-Supervised Learning with Adaptive Frequency-Time Attention Transformer for Seizure Prediction and Classification

**DOI:** 10.3390/brainsci15040382

**Published:** 2025-04-07

**Authors:** Yajin Huang, Yuncan Chen, Shimin Xu, Dongyan Wu, Xunyi Wu

**Affiliations:** Department of Neurology, Huashan Hospital, Fudan University, Shanghai 200040, China; huangyaajin@163.com (Y.H.); 22111220035@m.fudan.edu.cn (Y.C.); 23111220036@m.fudan.edu.cn (S.X.); dongyanwu@fudan.edu.cn (D.W.)

**Keywords:** epilepsy, transformer, self-supervised, attention

## Abstract

Background: In deep learning-based epilepsy prediction and classification, enhancing the extraction of electroencephalogram (EEG) features is crucial for improving model accuracy. Traditional supervised learning methods rely on large, detailed annotated datasets, limiting the feasibility of large-scale training. Recently, self-supervised learning approaches using masking-and-reconstruction strategies have emerged, reducing dependence on labeled data. However, these methods are vulnerable to inherent noise and signal degradation in EEG data, which diminishes feature extraction robustness and overall model performance. Methods: In this study, we proposed a self-supervised learning Transformer network enhanced with Adaptive Frequency-Time Attention (AFTA) for learning robust EEG feature representations from unlabeled data, utilizing a masking-and-reconstruction framework. Specifically, we pretrained the Transformer network using a self-supervised learning approach, and subsequently fine-tuned the pretrained model for downstream tasks like seizure prediction and classification. To mitigate the impact of inherent noise in EEG signals and enhance feature extraction capabilities, we incorporated AFTA into the Transformer architecture. AFTA incorporates an Adaptive Frequency Filtering Module (AFFM) to perform adaptive global and local filtering in the frequency domain. This module was then integrated with temporal attention mechanisms, enhancing the model’s self-supervised learning capabilities. Result: Our method achieved exceptional performance in EEG analysis tasks. Our method consistently outperformed state-of-the-art approaches across TUSZ, TUAB, and TUEV datasets, achieving the highest AUROC (0.891), balanced accuracy (0.8002), weighted F1-score (0.8038), and Cohen’s kappa (0.6089). These results validate its robustness, generalization, and effectiveness in seizure detection and classification tasks on diverse EEG datasets.

## 1. Introduction

Epilepsy is a common neurological disorder that affects approximately 50 million people worldwide [1]. Patients with epilepsy experience recurrent seizures, which may lead to injuries, suffocation, or even death [2,3]. Seizure detection helps accurately localize the epileptogenic zone (EZ), the brain region responsible for initiating seizures. Surgical resection of the EZ can render some patients seizure-free [4,5]. Epilepsy can occur at any age, and early detection is crucial for preventing further damage during physiological development and improving patients’ life expectancy [6]. An electroencephalogram (EEG) is an objective method of recording brain activity using scalp electrodes. It can reveal abnormal brain activity and is widely used to study neuronal activity patterns associated with brain disorders [7]. Deep learning algorithms have demonstrated potential in predicting seizures, offering the promise of improving quality of life for patients [8]. However, current deep learning-based methods for epileptic seizure detection primarily rely on supervised learning strategies with datasets manually annotated by professionals, which poses several challenges in practical applications [9,10,11]. First, acquiring large-scale, high-quality annotated data is not only time-consuming and labor-intensive, but also constrained by the scarcity of experts and the high annotation cost [12]. Second, existing supervised training methods are often tailored to specific types of seizures, lacking generalization ability across diverse seizure patterns, which limits the generalizability and robustness of the algorithms. Additionally, existing methods fail to fully utilize the vast amount of unlabeled EEG data and face computational complexity challenges when processing large-scale, high-dimensional EEG signals [13]. To address these issues, this paper proposes a self-supervised learning-based Transformer network for EEG signal analysis and epileptic seizure detection. This method reduces the reliance on annotated data and enhances the generalization ability for complex seizure patterns, providing technical support for early detection and precise prediction of epilepsy.

In recent years, deep learning (DL) methods have made significant strides in EEG analysis, with applications extending to epilepsy detection and classification. Convolutional neural networks (CNNs) have been widely employed for feature extraction due to their effectiveness in modeling spatial and temporal patterns of EEG signals. Notable examples include EEGNet, a lightweight architecture designed for brain–computer interface (BCI) applications [9], SCCNet, which integrates spatial and temporal convolutional modules to optimize spectral feature extraction [14], and FBCNet, which introduces spectral filtering during the initial feature extraction stage to enhance performance [15]. Beyond CNNs, graph neural networks (GNNs) have shown promise in capturing the complex dependencies inherent in EEG data. Models like Time2Graph and SimTSC use graph structures to represent EEG signals, achieving outstanding results in tasks such as epilepsy diagnosis and emotion recognition [10,11]. However, the reliance on predefined sensor topologies limits the generalizability of GNN-based approaches. More recently, Transformer-based models have attracted attention for their ability to capture long-term dependencies and global contextual features. For instance, EEGformer combines convolutional layers and Transformers to learn spatiotemporal features [16], MEET employs multi-band analysis to decode brain states [17], and ESTformer leverages spatiotemporal dependencies to enhance low-resolution EEG data [18]. Despite significant advancements, most methods still rely on supervised learning, which requires costly and time-intensive large-scale annotated datasets. Furthermore, their reliance on task-specific annotations limits generalization and poses challenges in handling multi-task demands and complex clinical diagnoses.

Self-supervised learning (SSL) has emerged as a powerful tool in EEG signal analysis, addressing the challenges posed by limited labeled datasets. Self-supervised graph neural networks for improving EEG seizure analysis introduce a framework based on GNNs, where node prediction and edge reconstruction tasks are used to extract spatiotemporal dependencies among EEG channels, laying the foundation for utilizing graph structures in EEG analysis [12]. Building on this, the BIOT [13] model extends SSL to cross-dataset learning, leveraging channel segmentation and reassembly into “sentence”-like structures to handle heterogeneous biosignals and mismatched channel setups. This approach bridges the gap between fixed-structure GNNs and real-world EEG variability, emphasizing flexibility [13]. Further enhancing universal EEG representation, EEGPT [19] adopts a Transformer-based architecture with masked reconstruction and temporal alignment tasks, achieving robust performance across multi-task scenarios. This method integrates insights from BIOT’s cross-domain focus but scales to broader, unified tasks using high-capacity Transformers.

While the aforementioned methods primarily achieve self-supervised training for EEG signals and enhance EEG feature extraction through Transformers, the inherent noise and data corruption in EEG signals can interfere with self-supervised training. This interference not only destabilizes the training process but also risks encoding noise into the learned representations, thereby degrading the model’s learning quality. To mitigate the impact of noise and capture stable and interpretable features, VQ-MTM [20] introduces random projection and phase alignment, refining the extraction of semantic units for noisy EEG signals. However, this approach does not fully address the issue of noise being encoded into the representations.

In this paper, we propose a self-supervised Transformer network with Adaptive Frequency-Time Attention (AFTA) for EEG signal analysis, utilizing a masking-and-reconstruction framework. This design directly addresses three critical limitations in EEG self-supervised learning identified in recent studies [21]: (1) spectral contamination from non-stationary noise (e.g., 20–60 Hz muscle artifacts present in 68% of scalp EEG recordings [22]) that corrupts learned representations; (2) temporal fragmentation caused by existing Transformers’ inability to model multi-scale dynamics spanning milliseconds (interictal spikes) to minutes (preictal shifts) [23]; and (3) domain adaptation gaps due to fixed frequency filters that fail to adapt to patient-specific spectral patterns [24]. Building upon the standard Transformer architecture, we implement a partial masking approach that selectively occludes portions of the original EEG signals [13,19]. Additionally, we integrate AFTA into the Transformer’s encoding to enhance the model’s ability to infer missing information from the unmasked data. The AFTA employs an adaptive filtering approach [25] and integrates with the Transformer’s self-attention mechanism to mitigate the impact of inherent noise in EEG signals and improve feature extraction capabilities. In the AFTA, the Adaptive Frequency Filtering Technique (AFFT) dynamically adjusts frequency filters based on the spectral properties of the input signal, enabling adaptive noise suppression and preservation of critical frequency components. This filtered output is then fed into the self-attention mechanism, which captures temporal dependencies and contextual relationships within the signal. The fusion of AFFT and self-attention allows the module to simultaneously model frequency-domain adaptability and time-domain dynamics, effectively mitigating non-stationary noise (e.g., muscle artifacts) while enhancing the extraction of discriminative EEG features.

The main contributions of this paper are as follows:

- A self-supervised learning Transformer network with AFTA is created for learning robust EEG feature representations from unlabeled data. The pretrained model is then fine-tuned and successfully applied to downstream tasks such as seizure prediction and classification.
- A novel AFTA is integrated into the Transformer architecture. This mechanism mitigates EEG noise and data corruption by applying adaptive global and local frequency-domain filtering and fusing the extracted features with temporal attention.
- The AFFM is designed to integrate into the Transformer architecture, enabling dynamic frequency-domain filtering to capture both global and local features. AFFM enhances the extraction of task-relevant EEG features without requiring additional hyperparameter tuning, thereby improving the model’s generalizability and practicality.
- The effectiveness of the proposed method is validated across three Temple datasets, demonstrating its capability in seizure prediction, classification, and comprehensive EEG signal analysis. Extensive evaluations on diverse tasks confirm the robustness and generalization ability of the model, showcasing its suitability for various EEG-related applications.

## 2. Related Work

### 2.1. Deep Learning-Based EEG Analysis

EEG signals inherently contain noise, such as ocular and muscular artifacts, which pose significant challenges to classification tasks. Traditional methods, such as regression and filtering, can partially mitigate noise but often lead to the loss of valuable information [26]. Advanced denoising techniques, including blind source separation [27] and wavelet transform [28], have been proposed for signal enhancement; however, the complexity of parameter selection remains a major limitation. In recent years, deep learning has gained wide application in EEG prediction and classification tasks, employing CNNs, recurrent neural networks (RNNs), attention mechanisms, GNNs, and Transformers. These methods aim to automatically extract complex features from EEG data to improve the accuracy and robustness of signal analysis.

CNNs are widely employed for feature extraction in EEG analysis due to their effectiveness in capturing spatial and temporal patterns. Among these, EEGNet stands out as a classical lightweight model specifically designed for BCI applications, offering compactness and efficiency [9]. SCCNet further advances this approach by integrating spatial and temporal convolutional modules, optimizing the extraction of spectral features [14]. Building on this, FBCNet introduces spectral filtering during the initial feature extraction stage, leading to significant performance improvements [15].

GNNs have also demonstrated their ability to model complex dependencies in EEG signals. For example, Time2Graph converts time-series data into graphs using time-aware shapelets, providing a novel representation of temporal patterns [10]. Time2Graph+ extends this approach by incorporating temporal attention mechanisms, further refining the graph representation [29]. SimTSC constructs graph edges based on time-series similarity and leverages node embeddings for classification [11]. These GNN-based methods have achieved promising results in various tasks, such as emotion recognition [30], epilepsy diagnosis [31], and sleep stage classification [32]. However, their reliance on predefined sensor topologies poses a limitation to their generalizability across diverse EEG datasets.

In recent years, Transformer networks have emerged as a powerful tool for EEG analysis, leveraging global attention mechanisms to capture long-term dependencies and address complex nonlinear dynamics. For instance, the EEG Conformer model effectively combines CNNs and Transformers, enabling the learning of both local and global features and achieving state-of-the-art performance in classification tasks [33]. Moreover, Zhu et al.’s work [34] on epileptic seizure prediction via multidimensional transformer and recurrent neural network fusion emphasizes the significance of Transformers. Similarly, EEGformer employs convolution for channel feature extraction and Transformers for spatiotemporal modeling, excelling in tasks such as emotion recognition [16]. MEET utilizes multi-band analysis to decode brain states, highlighting its utility in BCI applications [17]. Building on these advances, Xiang et al. [35] proposed a synchronization-based graph spatio-temporal attention network (SGSTAN) that jointly models dynamic brain network propagation and critical preictal time dependencies, achieving state-of-the-art seizure prediction performance on clinical EEG data. Meanwhile, ESTformer capitalizes on spatiotemporal dependencies to perform super-resolution reconstruction, significantly improving the quality of low-resolution EEG data [18]. Although existing Transformer-based methods have achieved notable success in EEG analysis, they still face significant limitations in handling the inherent noise and variability of EEG signals. To address these challenges, we implement an AFTA mechanism within the Transformer framework which leverages adaptive frequency-domain and time-domain feature extraction to further enhance the modeling capability and robustness of EEG signal analysis.

### 2.2. Self-Supervised Learning for EEG

EEG signals are inherently characterized by a low signal-to-noise ratio (SNR), substantial inter-individual variability, and task-dependent dynamics, which present significant challenges for both research and practical applications [36]. In modern machine learning, the pretraining-finetuning paradigm has revolutionized natural language processing (NLP) [37,38] and computer vision [39,40,41], providing a foundation for leveraging large-scale unlabeled data. Inspired by these advances, self-supervised learning (SSL) has been introduced to EEG analysis, enabling efficient use of large-scale unlabeled EEG datasets and significantly improving model performance [12,42,43]. Furthermore, progress in SSL for general time-series data [44,45,46] has provided new opportunities to enhance EEG data analysis.

Several SSL-based methods have advanced EEG analysis with unique approaches. BENDR pioneered SSL for EEG by combining masked autoencoders and contrastive learning to extract local temporal features via a transformer decoder [47]. EEG2VEC further leveraged contrastive and reconstruction losses to capture both local and global features using convolutional and transformer networks, proving effective for downstream tasks [48]. Similarly, Xiao et al. [49] proposed a self-supervised learning method for EEG-based seizure detection, which utilizes an attention mechanism-based Transformer to extract global dependencies and effectively addresses the issue of insufficient annotated data. Falck et al. extended SimCLR to time-series data, enabling channel feature extraction and achieving 85.12% accuracy on the Sleep-EDF dataset, though limited to short 20-s EEG tasks [43,50]. Addressing cross-dataset challenges, BIOT [13] introduced fixed-length channel tokenization, rearranging segments into “long sentences”, improving performance by 4% on the CHB-MIT seizure detection task [13,51]. Similarly, LaBraM enhanced cross-dataset learning by segmenting EEG into channel patches, using vector-quantized spectrum prediction for neural tokenizer training, and pretraining transformers to predict masked patches’ original codes [52]. These innovations have collectively expanded the potential of self-supervised learning for robust EEG feature extraction.

Despite these advancements, several challenges persist. The low SNR of EEG signals and the inherent complexity of brain activity during tasks make learning abstract features through masked autoencoders particularly difficult [47]. Moreover, inconsistencies in sampling rates and electrode placements across different EEG acquisition devices pose additional obstacles, limiting the ability of convolutional encoders to decouple channel-specific dependencies and affecting the robustness and scalability of SSL models [13]. Addressing these issues remains critical for advancing the application of self-supervised learning in EEG analysis.

## 3. Materials and Methods

### 3.1. Principles of Self-Supervised Learning for EEG

**Figure 1:**
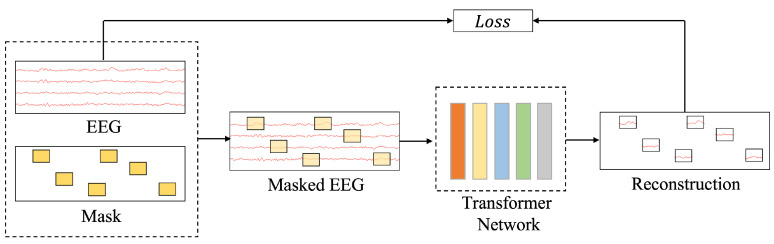
The principles of self-supervised learning for EEG. This figure shows a self-supervised learning workflow for EEG data: raw EEG signals are masked (yellow blocks in “Mask”) to create masked EEG, processed by a Transformer network (colored bars), and reconstructed (signal snippets in “Reconstruction”). The “Loss” evaluates reconstruction accuracy, driving the learning process.

The core of EEG self-supervised learning (EEG-SSL) lies in learning high-quality feature representations from unlabeled EEG data. The principle of self-supervised learning for EEG involves masking a portion of the original EEG signals to generate partially occluded signals (Masked EEG), as shown in Figure 1. A Transformer network is then used to encode and reconstruct the masked EEG signals, thereby learning the intrinsic features of the data. The model calculates the loss by comparing the reconstructed signals with the original ones, using this as the optimization objective to enhance its representation of EEG features progressively. This approach does not require extensive labeled data but leverages the inherent structure of EEG signals to achieve effective feature learning, providing high-quality representations for downstream tasks such as classification or anomaly detection. EEG-SSL employs a dual self-supervised learning framework that combines masked modeling and spatiotemporal representation alignment to extract universal and robust spatiotemporal features from EEG signals [12,13,19].

**Figure 2:**
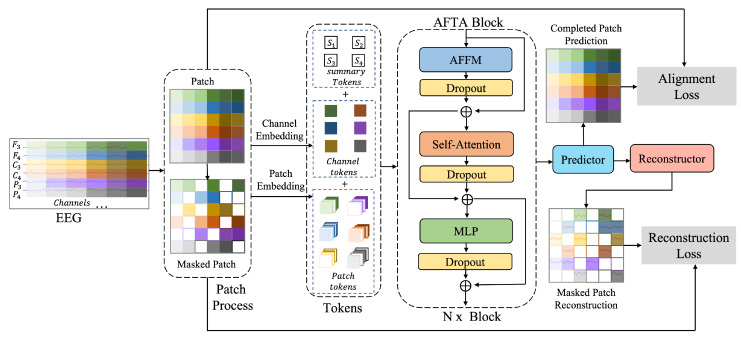
The Transformer framework for self-supervised EEG learning. This framework consists of an encoder and a decoder. In the encoder, the raw EEG signals are divided into patches, with some patches being masked (Masked Patch). Both the masked and unmasked patches are processed through channel embedding and patch embedding to generate channel tokens and patch tokens, which are combined with summary tokens and input into N Adaptive Frequency-Time Attention (AFTA) Blocks. The AFTA Block, as the core module of the framework, consists of the Adaptive Frequency-Time Fusion Module (AFFM), the Self-Attention Module, and a Multi-Layer Perceptron (MLP). In the decoder, the predictor aligns the predictions for the masked patches with the original signals for consistency, while the reconstructor further reconstructs the masked patches from the predicted features and performs reverse alignment, completing the self-supervised learning process.

The frameworks of our method are shown in Figure 2. Firstly, EEG-SSL applies masking to the input EEG signals, where a portion of the temporal patches and channel patches is randomly masked to construct a pretext task. The unmasked signals are processed through an encoder to extract feature representations and generate global spatiotemporal features. Following BIOT [13] and EEGPT [19], the model then uses a predictor to predict the features of the masked patches and a reconstructor to rebuild the masked EEG signals. The reconstructed signals are compared with the original masked signals using the frequency-balanced mean squared error (MSE) loss proposed by BIOT [13] to optimize reconstruction accuracy:

(1) $$L_{R}=\frac{1}{\left|M\right|}\sum_{(i,j)\in M}\left|\right|{\mathrm{rec}}_{i,j}-{\mathrm{pi}}_{i,j}{\left|\right|}^{2}$$

where *M* denotes the set of masked patches, ${\mathrm{rec}}_{i,j}$ represents the reconstructed signals, and ${\mathrm{pi}}_{i,j}$ is the original signal.

Furthermore, EEG-SSL incorporates a spatiotemporal representation alignment mechanism based on the Riemannian manifold alignment principles in EEGPT [19], by aligning the features generated by the encoder with the target features generated by a momentum encoder. This enhances the global consistency of the encoded features. The alignment loss follows the contrastive geometry framework proposed by [19] and is defined as:

(2) $$L_{A}=-\frac{1}{N}\sum_{j=1}^{N}\left|\right|{\mathrm{pred}}_{j}-\mathrm{LN}\left({\mathrm{menc}}_{j}\right){\left|\right|}^{2}$$

where ${\mathrm{pred}}_{j}$ represents the output features of the predictor, ${\mathrm{menc}}_{j}$ represents the target features generated by the momentum encoder, and LN(·) denotes layer normalization.

EEG-SSL optimizes its learning process by combining reconstruction loss and alignment loss:

(3) $$L=L_{A}+L_{R}$$

After completing the self-supervised learning process, EEG-SSL can be fine-tuned for specific downstream tasks, such as seizure prediction and seizure type classification.

### 3.2. Self-Supervised Learning Transformer Networker

Based on BIOT [13] and EEGPT [19], we propose a self-supervised learning Transformer network with AFTA module for EEG signal analysis using a masking-and-reconstruction framework, as shown in Figure 2. Following EEGPT [19], EEG signals are divided into spatiotemporal patches, with 50% of temporal and 80% of channel patches masked to encourage spatiotemporal learning. Spatiotemporal patches are local segments obtained by dividing EEG signals along the temporal and spatial dimensions. In the temporal dimension, the signals are divided into fixed-length time windows (e.g., 1 s), while in the spatial dimension, they are grouped based on different channels (e.g., F3, C4). This partitioning method allows for the concurrent analysis of temporal changes and spatial patterns in signals, laying the groundwork for further feature extraction and self-supervised learning.

Embedded patches, combined with channel and positional encodings, are processed by a Transformer encoder to extract contextual features. The Reconstructor integrates encoder features and predictor outputs to reconstruct masked patches. A reconstruction loss, calculated as mean squared error, optimizes the model to recover masked signals, enabling robust representation learning from unlabeled EEG data.

#### 3.2.1. EEG Patch Processing

EEG signals, represented as multichannel time-series data $X\in R^{C\times T}$, where *C* denotes the number of channels and *T* represents the temporal length, are partitioned into fixed-size time-space patches following the neurophysiologically-grounded scheme initially proposed in BIOT [13] and subsequently refined with Riemannian manifold projections in EEGPT [19]. The design of spatio-temporal patches enables the model to simultaneously process the temporal dependencies and spatial topology of EEG signals. For instance, in epileptic seizure prediction tasks, spatio-temporal patches can capture abnormal discharge patterns in specific brain regions during the prerectal phase, thereby improving prediction accuracy. Each patch captures a specific temporal segment and spatiotemporal receptive field, adapting principles from [13,19]: the temporal segmentation employs overlapping 200 ms windows (inherited from [13]), while the spatial grouping integrates hemispheric symmetry priors (following [19], Section 3.2) with our novel dynamic multi-scale kernel design.

Formally, a patch $p_{i,j}$ is defined as:

(4) $$p_{i,j}=X\left[i,j:j+d\right],\;i\in \left\{1,2,\ldots ,C\right\},\;j\in \left\{1,2,\ldots ,T/d\right\}$$

where *d* represents the temporal length of each patch, and T/d denotes the total number of temporal partitions.

A masking strategy is applied, where 50% of the temporal patches and 80% of the channel patches are randomly masked to form a masked set M and an unmasked set $\bar{M}$. This masking forces the model to infer global spatiotemporal relationships from the unmasked patches, improving its understanding of the signal structure.

A masking strategy is applied, where 50% of the temporal patches and 80% of the channel patches are randomly masked to form a masked set M and an unmasked set $\bar{M}$. This masking forces the model to infer global spatiotemporal relationships from the unmasked patches, enhancing its understanding of the signal structure. We justify this approach based on prior studies (e.g., [13,19]), which demonstrate that selective masking increases the model’s ability to capture complex dependencies in EEG signals by encouraging it to reconstruct missing data from contextual information in the unmasked patches.

#### 3.2.2. Feature Embedding

Each patch is linearly mapped to a feature embedding and combined with channel embeddings and temporal positional encodings to encode spatial and temporal information [13]:

(5) $${\mathrm{token}}_{i,j}=W_{p}^{⊤}p_{i,j}+b_{p}+\zeta_{i}+\pi_{j}$$

where $W_{p}$ and $b_{p}$ are the parameters of the linear embedding layer; $\zeta_{i}$ denotes the channel embedding to differentiate EEG channels; and $\pi_{j}$ represents the temporal positional encoding, which captures sequential information.

Additionally, learnable summary tokens $s_{k}$ are introduced as standalone global tokens. These tokens are concatenated to the sequence of patch tokens, enabling the model to encode both local patch-level features and global contextual information [13]:

(6) $$\mathrm{tokens}={\left\{{\mathrm{token}}_{i,j}\right\}}_{i,j}\cup \left\{s_{k}\right\}$$

For masked patches, only the positional encodings and channel embeddings are provided, while the actual signal values are omitted. This encourages the model to infer the missing data based on the relationships among unmasked patches and the global context provided by the summary tokens [13,19]. Unmasked patches include their full signal values, allowing the model to learn both detailed local features and broader spatiotemporal dependencies.

#### 3.2.3. Transformer Encoder

The embedded patch features are fed into the Transformer encoder, which consists of multiple layers of self-attention and feed-forward networks (FFNs). The encoder extracts global spatiotemporal features from the unmasked patches [13]:

(7) $${\mathrm{enc}}_{j}=\mathrm{ENC}\left({\mathrm{token}}_{i,j}\right)$$

where ENC denotes the computation process of the Transformer encoder. The attention mechanism allows the masked patches to interact with the unmasked ones, capturing the contextual features required to reconstruct the masked information.

The features extracted by the encoder, ${\mathrm{enc}}_{j}$, are passed to subsequent modules for signal reconstruction.

#### 3.2.4. Reconstructor and Signal Reconstruction

The reconstructor combines the encoder output features ${\mathrm{enc}}_{j}$ and the predictor-generated features ${\mathrm{pred}}_{j}$, along with the temporal positional encoding ${\mathrm{pos}}_{j}$, to reconstruct the masked patches [13]:

(8) $${\left\{{\mathrm{rec}}_{i,j}\right\}}_{(i,j)\in M}=\mathrm{REC}\left(\left\{{\mathrm{enc}}_{j}+{\mathrm{pos}}_{j}\right\}\cup \left\{{\mathrm{pred}}_{j}+{\mathrm{pos}}_{j}\right\}\right)$$

where REC denotes the computation process of the reconstruction module.

During reconstruction, the predicted features of the masked patches are integrated with the global features of the unmasked patches, allowing the model to learn the spatiotemporal relationships necessary for signal recovery. A skip connection is introduced, directly passing the encoder features to the reconstructor, ensuring consistency and accelerating convergence.

The reconstructed signal ${\mathrm{rec}}_{i,j}$ is compared with the original masked signal ${\mathrm{pi}}_{i,j}$, and the reconstruction loss is computed using the mean squared error (MSE) [13]. This masking and reconstruction mechanism enables the model to learn informative feature representations from unlabeled EEG data.

(9) $$L_{R}=\frac{1}{\left|M\right|}\sum_{(i,j)\in M}\left|\right|{\mathrm{rec}}_{i,j}-{\mathrm{pi}}_{i,j}{\left|\right|}^{2}$$

### 3.3. Adaptive Frequency-Time Attention Block

**Figure 3:**
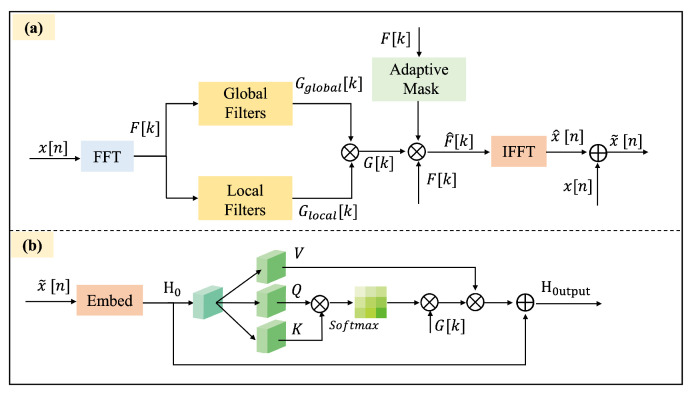
Structure of the adaptive frequency-time attention block. (**a**) Adaptive frequency filtering module: Input x[n] is transformed via FFT to F[k], filtered by global ($G_{\mathrm{global}}\left[k\right]$) and local ($G_{\mathrm{local}}\left[k\right]$) filters with an adaptive mask, and reconstructed via IFFT to $\tilde{x}\left[n\right]$, combined with x[n] to form $\hat{x}\left[n\right]$. (**b**) Frequency-time attention: $\tilde{x}\left[n\right]$ is embedded into $H_{0}$, processed with query (*Q*) and key (*K*) via softmax and attention weights G[k] to output $H_{\mathrm{output}}$.

The AFTA block integrates frequency-time domain transformation, adaptive filtering module, and frequency-time domain fusion mechanism to improve the modeling capability of complex signals, as shown in Figure 3. Drawing on TSLANet [25], our innovation in AFFM is integrating adaptive global-local filtering with the self-attention. The framework leverages dynamic frequency filtering and multi-feature collaborative modeling to effectively support both classification and prediction tasks.

#### 3.3.1. Adaptive Frequency Filtering Module

The core of the AFFM lies in dynamically adjusting frequency-domain filters to accommodate varying signal characteristics and extract task-relevant frequency components [26], as shown in Figure 3. This module encompasses the following steps:

Global and local frequency-domain dynamic filters. The input signal x[n] is transformed into its frequency-domain representation:

(10) F[k]=FFT(x[n]),

where FFT denotes the Fast Fourier Transform.

To achieve finer adjustments in the frequency domain, the module introduces both global and local complex dynamic filters:

(11) $$G_{\mathrm{global}}\left[k\right]=\sigma \left(W_{\mathrm{global}}\left[k\right]\right),$$

(12) $$G_{\mathrm{local}}\left[k\right]=\sigma \left(W_{\mathrm{local}}\left[k\right]\right),$$

where σ(·) is the sigmoid activation function, and $W_{\mathrm{global}}\left[k\right]$ and $W_{\mathrm{local}}\left[k\right]$ are learnable weights for each frequency component in the global and local filters, respectively. The global filter adjusts the overall frequency characteristics, while the local filter provides fine-grained adjustments for significant frequency components.

Combining global and local filters: the weights of the global and local filters are combined through complex multiplication and addition:

(13) $$G\left[k\right]=G_{\mathrm{global}}\left[k\right]\cdot G_{\mathrm{local}}\left[k\right],$$

resulting in a combined filter G[k] that accounts for both global frequency characteristics and local frequency variations.

Adaptive frequency-domain masking: a dynamic threshold θ is introduced to select significant frequency components [25]:

(14) $$\hat{F}\left[k\right]=F\left[k\right]\cdot \mathrm{AdaptiveMask}\left(F\left[k\right],\theta \right),$$

where AdaptiveMask(F[k],θ) generates an adaptive mask based on the energy distribution of the frequency-domain signal. Specifically, the power spectral density is calculated as:

(15) $$P\left[k\right]={\left|F\left[k\right]\right|}^{2},$$

and the adaptive mask is generated by comparing the normalized energy against the threshold:

(16) $$\mathrm{AdaptiveMask}\left(F\left[k\right],\theta \right)=\left(\frac{P[k]}{\mathrm{median}(P[k])+\epsilon }>\theta \right),$$

where ϵ is a small constant to prevent division by zero. The mask AdaptiveMask(F[k],θ) selectively retains significant frequency components, effectively suppressing noise and irrelevant frequencies.

The threshold θ is optimized by minimizing the following loss function:

(17) $$L_{\mathrm{adaptive}}=L_{\mathrm{task}}+\lambda {∥\theta ∥}^{2},$$

where $L_{\mathrm{task}}$ represents the task-related loss, and λ is a regularization coefficient to prevent overfitting.

To combine the global and local filters with the mask, the combined filter G[k] is applied to the masked frequency-domain signal:

(18) $$\hat{F}\left[k\right]=F\left[k\right]\cdot \mathrm{AdaptiveMask}\left(F\left[k\right],\theta \right)\cdot G\left[k\right],$$

resulting in the filtered frequency-domain signal $\hat{F}\left[k\right]$.

Inverse FFT and time-domain signal fusion is achieved via the transformation of the filtered frequency-domain signal $\hat{F}\left[k\right]$ back to the time domain using the inverse Fourier transform:

(19) $$\hat{x}\left[n\right]=\mathrm{IFFT}\left(\hat{F}\left[k\right]\right),$$

where IFFT denotes the Inverse Fast Fourier Transform.

Subsequently, the filtered time-domain signal $\hat{x}\left[n\right]$ is fused with the original time-domain signal x[n] as follows:

(20) $$\tilde{x}\left[n\right]=\alpha \cdot x\left[n\right]+\left(1-\alpha \right)\cdot \hat{x}\left[n\right],$$

where α is a learnable parameter that balances the contributions of the original and filtered signals.

#### 3.3.2. Frequency-Time Attention

The output of the adaptive filtering module is integrated with a self-attention mechanism to achieve collaborative modeling of frequency and time-domain features, as shown in Figure 3, implemented as follows:

In high-dimensional signal embedding, the fused signal $\tilde{x}\left[n\right]$ is mapped to a high-dimensional space using an embedding function:

(21) $$H_{0}=\mathrm{Embed}\left(\tilde{x}\left[n\right]\right).$$

To achieve frequency-aware self-attention, frequency-domain weights are incorporated into the self-attention mechanism to focus on task-relevant frequency components:

(22) $$A=\mathrm{softmax}\left(\frac{QK^{⊤}}{\sqrt{d_{k}}}\right),\;A_{f}=A\cdot \mathrm{diag}\left(G\left[k\right]\right)\cdot V,$$

where $A_{f}$ represents the frequency-enhanced attention distribution, *Q* and *K* are the query and key matrices, *V* is the value input features, and $d_{k}$ is the feature dimension.

Enhanced residual connections are incorporated to preserve the integrity of the original signal:

(23) $$H_{\mathrm{output}}=\mathrm{LayerNorm}\left(H_{\mathrm{attn}}+\tilde{H}+\mathrm{FC}\left(H_{\mathrm{attn}}\right)\right),$$

where $H_{\mathrm{attn}}$ is the output of the self-attention module, and $\tilde{H}$ is the embedding of the fused signal. FC denotes a fully connected layer, and LayerNorm refers to layer normalization.

### 3.4. Downstream Tasks

In this work, we design the loss functions for the classification and prediction of seizures and apply the self-supervised pre-trained network to the downstream tasks to further validate the effectiveness of our approach [20]. Specifically, we define two tasks, the seizure classification task and the seizure prediction task, as outlined below.

#### 3.4.1. Seizure Classification Task

The objective is to maximize classification accuracy, with the loss defined as:

(24) $$L_{\mathrm{classification}}=-\sum_{i}y_{i}\log\left({\hat{y}}_{i}\right),$$

where $y_{i}$ and ${\hat{y}}_{i}$ denote the ground truth and predicted class probabilities, respectively.

#### 3.4.2. Seizure Prediction Task

The objective is to minimize prediction error, using the mean squared error (MSE) loss:

(25) $$L_{\mathrm{prediction}}=\frac{1}{N}\sum_{i=1}^{N}{\left(y_{i}-{\hat{y}}_{i}\right)}^{2},$$

where $y_{i}$ and ${\hat{y}}_{i}$ are the ground truth and predicted values, respectively.

### 3.5. Data and Processing

#### 3.5.1. Pretrain Data and Processing

In the pretraining phase, we selected multiple EEG datasets, including the emotion classification dataset SEED [53], the motor execution (ME) dataset PhysioMI [54], and the steady-state visual evoked potential (SSVEP) dataset TSU [55], for model pretraining.

We uniformly preprocessed these EEG datasets to create a combined pretraining dataset. First, specific EEG channels were selected, and irrelevant or noisy channels were removed to ensure consistent channel ordering across all datasets. Next, band-pass filtering (0.1–75 Hz) and a 50 Hz notch filter were applied to eliminate low-frequency drift and power line interference, followed by resampling the data to 200 Hz. To enhance data diversity, we employed random reflection and scaling for data augmentation. Subsequently, temporal interpolation was used to standardize the length of EEG sequences to a fixed duration of 4 s, and signal amplitudes were converted to μV. Finally, the preprocessed data were transformed into two-dimensional tensor formats and saved to designated directories, facilitating the subsequent training and evaluation of deep learning models.

#### 3.5.2. Downstream Task Data and Processing

In downstream applications, we utilized four comprehensive EEG datasets developed by Temple University Hospital [56]—TUAB (v3.0.1), TUSZ (v2.0.3), TUEV (v2.0.1), and CHB-MIT [57]—to evaluate the effectiveness of self-supervised pretraining in epilepsy prediction, classification, and EEG signal analysis. TUAB includes over 3000 EEG records totaling approximately 1500 h of continuous monitoring. It covers various clinical diagnoses such as epilepsy, brain injuries, and comas, with each record captured using 21 electrodes to ensure high spatial resolution. This dataset is extensively used for developing deep learning models, researching feature extraction methods, and constructing real-time monitoring systems. TUAB is characterized by its focus on general EEG abnormality detection, providing a diverse range of clinical EEG recordings labeled as normal or abnormal. TUSZ focuses on epilepsy-related EEG data, containing around 6500 h of recordings and over 26,000 annotated seizure events. These annotations, performed by expert neurologists, encompass multiple types of epilepsy, providing rich temporal and electrophysiological features essential for training and evaluating seizure detection and prediction algorithms. TUSZ is distinguished by its extensive collection of annotated seizure events, making it ideal for epilepsy-specific research. TUEV offers approximately 1800 h of high-quality EEG recordings from a wide range of clinical scenarios, including sleep disorders and attention deficit hyperactivity disorder (ADHD). Each record is equipped with 21 electrodes and includes detailed annotations of events, clinical diagnoses, and patient information, making TUEV a critical resource for validating and assessing the accuracy, stability, and generalizability of automated EEG analysis algorithms. TUEV is notable for its detailed annotations of various EEG events beyond seizures, such as spikes and sharp waves, offering insights into diverse neurological phenomena. A comparative introduction to the four different datasets is shown in Table 1.

In this study, we uniformly preprocessed the TUAB (v3.0.1) and TUEV (v2.0.1) EEG datasets from Temple University Hospital. We selected specific EEG channels and removed irrelevant or noisy ones to maintain consistent channel ordering across datasets. Band-pass filtering (0.1–75 Hz) and a 50 Hz notch filter were applied to eliminate low-frequency drift and power line interference, followed by resampling to 200 Hz. Temporal interpolation standardized EEG sequence lengths to 4 s, and signal amplitudes were converted to μV.

When processing the TUSZ dataset, in addition to adhering to the fundamental preprocessing steps established for TUAB and TUEV, we specifically tailored the workflow for epilepsy detection and classification tasks. We segmented the EEG data into fixed-length segments (e.g., 12 s) to ensure that each segment contained only one type of epileptic seizure or non-epileptic activity. Furthermore, we selected 19 channels based on the standard 10–20 system and reordered the channels as necessary to maintain data consistency across all recordings. For label processing, we consolidated the original eight seizure types (FNSZ, GNSZ, SPSZ, CPSZ, ABSZ, TNSZ, TCSZ, MYSZ) into four categories: Combined Focal Non-Specific Seizure (CFSZ), Generalized Non-Specific Seizure (GNSZ), Absence Seizure (ABSZ), and Combined Tonic Seizure (CTSZ).

Furthermore, we conducted experiments on another epilepsy dataset, CHB-MIT [57]. This dataset consists of scalp EEG recordings from 23 pediatric patients with intractable seizures, resulting in 24 cases due to one patient having two records taken 1.5 years apart. It includes approximately 969 h of continuous EEG data, encompassing 173 seizure events. The recordings were sampled at 256 Hz with 16-bit resolution. The EEG data were collected using the international 10–20 system for electrode placement, primarily utilizing 23 channels, although some cases include 24 or 26 channels.

In this study, we uniformly preprocessed the CHB-MIT [57] EEG dataset to ensure consistency and quality of the data for seizure prediction and classification tasks. First, we selected 16 common EEG channels across all patients to maintain consistent channel ordering and excluded irrelevant or noisy channels. The raw EEG signals were then segmented into 4 s. To eliminate low-frequency drift and high-frequency noise, we applied a band-pass filter (1–40 Hz) and a 50 Hz notch filter to remove power line interference. The signals were resampled to 200 Hz for uniformity. Finally, the preprocessed data were labeled for seizure and non-seizure events, and sample balancing was performed to achieve a 1:1 ratio of positive to negative samples.

## 4. Experiments Setting

### 4.1. Experimental Environment

The experiment was conducted on a high-performance workstation equipped with an Intel i9-12900 CPU and 256 GB of Kingston RAM, running on the Ubuntu operating system. Model training utilized three NVIDIA RTX 3090 GPUs (NVIDIA Corporation, Santa Clara, CA, USA), each with 24 GB of VRAM, and CUDA technology was employed for acceleration. The development environment was built using Python 3.0 and PyTorch 2.10.

### 4.2. Implementation Details

The training setup for different datasets was carefully designed to optimize model performance and robustness. The AdamW [58] optimizer was used with a weight decay rate of 0.05 to mitigate overfitting. The training spanned 100 epochs (max epochs when loss no longer decreased), with a batch size of 120 (optimized for NVIDIA RTX 3090 GPU memory and input data), and a patch size of 200 (inspired by EEGPT [19]) for efficient feature extraction using GELU activation. A learning rate schedule with a linear warm-up phase was implemented, starting at an initial learning rate of $5\times 10^{-4}$ and increasing to its peak value over the first 15 epochs. Following this, the learning rate decayed progressively at a factor of 0.65 using a layer-wise decay mechanism, ensuring lower learning rates for deeper layers while accelerating feature learning in higher layers. A mask ratio of 0.5 and a dropout rate of 0.3 were applied to encourage robustness. Additionally, the model leveraged a codebook with 1024 entries, each having a dimensionality of 256, to enhance representation learning. These hyperparameter choices achieved a balance between computational efficiency and model performance.

The pretraining process employed a self-supervised masking-and-reconstruction framework to learn robust EEG representations from unlabeled data. We utilized a combination of EEG datasets (e.g., SEED, PhysioMI, TSU) preprocessed with bandpass filtering (0.1–75 Hz), notch filtering (50 Hz), and standardization to 4-s epochs. Adaptive masking was applied, occluding 50% of temporal patches and 80% of channels, encouraging the model to infer missing information. The Transformer architecture integrated Adaptive Frequency-Time Attention (AFTA) for joint frequency-time feature learning. Training used AdamW (initial $lr=5\times 10^{-4}$, weight decay = 0.05) with a cosine learning rate scheduler and batch size = 120 for 100 epochs. Dropout (rate = 0.3) and a codebook (1024 entries, 256 dimensions) enhanced robustness and representation learning. This approach achieved noise-invariant feature extraction, improving downstream task performance.

### 4.3. Evaluation Methodology

We employed four key metrics to evaluate the performance of our models: (1) balanced accuracy (BAC), which measures the mean recall across all classes and is suitable for both binary and multi-class classification; (2) AUROC, the area under the receiver operating characteristic curve, primarily used for binary classification; (3) weighted F1, the harmonic mean of precision and recall with class-specific weighting, ideal for multi-class classification; and (4) Cohen’s kappa, a statistic for measuring agreement between categorical variables. AUROC was used as the monitor score for binary classification, while Cohen’s kappa was applied for multi-class classification.

Balanced accuracy calculates the mean recall across all classes, providing a robust measure for datasets with imbalanced classes. It is applicable for both binary and multi-class classification tasks. Mathematically, BAC is defined as:

(26) $$\mathrm{BAC}=\frac{1}{C}\sum_{i=1}^{C}\frac{{\mathrm{TP}}_{i}}{{\mathrm{TP}}_{i}+{\mathrm{FN}}_{i}}$$

where *C* represents the number of classes, ${\mathrm{TP}}_{i}$ denotes the number of true positives for class *i*, and ${\mathrm{FN}}_{i}$ is the number of false negatives for class *i*.

AUROC evaluates the trade-off between the true positive rate (TPR) and the false positive rate (FPR) across different classification thresholds. It is primarily used for binary classification and is computed as the area under the ROC curve. The key components are:

(27) $$TPR=\frac{\mathrm{TP}}{\mathrm{TP}+\mathrm{FN}},\;FPR=\frac{\mathrm{FP}}{\mathrm{FP}+\mathrm{TN}}$$

where TP, FP, TN, and FN represent true positives, false positives, true negatives, and false negatives, respectively.

The weighted F1-score combines precision and recall in a harmonic mean, weighting each class by its support (i.e., the number of true samples in the class). This metric is particularly suitable for multi-class classification. It is defined as:

(28) $$\mathrm{Weighted}\;F1=\frac{1}{N}\sum_{i=1}^{C}\frac{2\cdot {\mathrm{Precision}}_{i}\cdot {\mathrm{Recall}}_{i}}{{\mathrm{Precision}}_{i}+{\mathrm{Recall}}_{i}}\cdot N_{i}$$

where $N_{i}$ is the number of true samples for class *i*, *N* is the total number of samples, and:

(29) $${\mathrm{Precision}}_{i}=\frac{{\mathrm{TP}}_{i}}{{\mathrm{TP}}_{i}+{\mathrm{FP}}_{i}},\;{\mathrm{Recall}}_{i}=\frac{{\mathrm{TP}}_{i}}{{\mathrm{TP}}_{i}+{\mathrm{FN}}_{i}}$$

Cohen’s kappa measures the agreement between two categorical variables while accounting for the possibility of chance agreement. It is defined as:

(30) $$\kappa =\frac{p_{o}-p_{e}}{1-p_{e}}$$

where $p_{o}$ is the observed agreement, calculated as the sum of the diagonal elements in the confusion matrix divided by the total number of observations, and $p_{e}$ is the expected agreement, derived from the marginal probabilities of each class.

In this study, AUROC was employed as the monitoring metric for binary classification tasks, while Cohen’s kappa was selected for multi-class classification to provide a comprehensive evaluation of inter-class agreement and model performance.

### 4.4. Baseline Methods

We use the same baselines from BIOT [13], which are fully fine-tuned models. We evaluated our proposed method against the state-of-the-art time series self-supervised learning and classification approaches, including DCRNN [12], TimesNet [44], PatchTST [45], SimMTM [46], MAE [40], SPaRCNet [59], ContraWR [60], CNN-T [61], FFCL [62], ST-T [63], BIOT [13], and EEGPT [19]. All baseline models were subjected to identical preprocessing steps and evaluated under the same experimental conditions as our proposed method. This consistency in preprocessing and experimental settings ensured a fair comparison, enabling an accurate assessment of the performance of our method relative to state-of-the-art self-supervised models for epileptic EEG classification and prediction.

## 5. Results

### 5.1. Performance Comparison

We conducted extensive comparative experiments and evaluated our proposed method against several state-of-the-art approaches on three public datasets. These experiments aimed to validate the effectiveness of our method in seizure prediction, classification, and EEG signal analysis.

In the experiments conducted on the TUSZ dataset, we compared our method with several state-of-the-art approaches to validate its effectiveness in seizure detection and classification tasks. As shown in Table 2, our method achieved the highest performance in both AUROC (0.891) and weighted F1-score (0.644) on 12-s segments from the TUSZ dataset. For seizure detection, our method achieved the highest AUROC of 0.891, surpassing all baseline methods, including VQ-MTM [20], which achieved 0.887, and PatchTST [45], which achieved 0.866. This demonstrates our method’s ability to effectively capture robust contextual representations for EEG signals. Similarly, for seizure classification, our approach achieved the highest weighted F1-score of 0.644, outperforming VQ-MTM (0.620) and PatchTST (0.607), and significantly improving over MAE [40] (0.592). These results validate the generalization and fine-grained classification capabilities of our method. Among the baseline methods, TimesNet [44] and PatchTST exhibited competitive performance, while SimMTM [46] consistently underperformed across both tasks due to its scalability limitations on large datasets like TUSZ.

In experiments on the TUAB dataset, our method achieved the highest balanced accuracy (0.8002±0.0025) and AUROC (0.8848±0.0038), surpassing all baselines, including EEGPT [19], which achieved 0.7983±0.0030 and 0.8718±0.0050, respectively, as shown in Table 3. These results demonstrate our method’s superior ability to handle class imbalance and extract robust, discriminative features for seizure detection. While EEGPT and BIOT [13] performed strongly, with BIOT achieving an AUROC of 0.8815±0.0043, methods like ST-T [63] and SPaRCNet [59] showed competitive but slightly lower performance. ContraWR [60] and CNN-T [61] exhibited lower metrics due to scalability and feature extraction limitations.

On the TUEV dataset, our method demonstrated competitive performance across all metrics compared to state-of-the-art methods, as shown in Table 4. It achieved the highest weighted F1-score (0.8038±0.0258), Cohen’s kappa (0.6089±0.0238), and balanced accuracy (0.5725±0.0182). Compared to EEGPT, which excelled in balanced accuracy and weighted F1 (0.781±0.0040), our method showed superior performance in Cohen’s kappa, indicating stronger agreement in predictions. BIOT [13] performed well in balanced accuracy (0.5281) and weighted F1 (0.7492), but it lagged behind both EEGPT and our method. While SPaRCNet [59] and ContraWR [60] achieved moderate results, CNN-T [61], FFCL [62], and ST-T [63] showed relatively lower performance.

On the CHB-MIT dataset, our method demonstrated superior performance across all metrics compared to state-of-the-art methods, as shown in Table 5. It achieved the highest balanced accuracy (0.8098±0.0206), weighted F1-score (0.5901±0.0274), and Cohen’s kappa (0.9454±0.0216). Compared to EEGPT [19], which excelled in balanced accuracy (0.7826±0.0137) and weighted F1 (0.5391±0.0173), our method showed further improvements in all metrics, particularly in Cohen’s kappa, indicating stronger agreement in predictions. BIOT [13] performed well in balanced accuracy (0.6814±0.0192) and Cohen’s kappa (0.8885±0.0136), but it lagged behind both EEGPT and our method in weighted F1 (0.2639±0.0103). While SPaRCNet [59] and CNN-T [61] achieved moderate results, ST-T [63] showed relatively lower performance across all metrics. These results highlight the robustness and effectiveness of our approach in seizure prediction and classification tasks on the CHB-MIT dataset.

Overall, the substantial improvements across all evaluation metrics validate the effectiveness, scalability, and adaptability of our proposed method for seizure detection and classification in EEG analysis.

### 5.2. Ablation Study for Pretraining Methods

The pretraining process employed a self-supervised masking-and-reconstruction framework to learn robust EEG representations from unlabeled data. We utilized a combination of EEG datasets (e.g., SEED, PhysioMI, TSU) preprocessed with bandpass filtering (0.1–75 Hz), notch filtering (50 Hz), and standardization to 4-s epochs. Adaptive masking was applied, occluding 50% of temporal patches and 80% of channels, encouraging the model to infer missing information. The Transformer architecture integrated Adaptive Frequency-Time Attention (AFTA) for joint frequency-time feature learning. Training used AdamW (initial lr = $5\times 10^{-4}$, weight decay = 0.05) with a cosine learning rate scheduler and batch size = 120 for 100 epochs. Dropout (rate = 0.3) and a codebook (1024 entries, 256 dimensions) enhanced robustness and representation learning.

**Figure 4:**
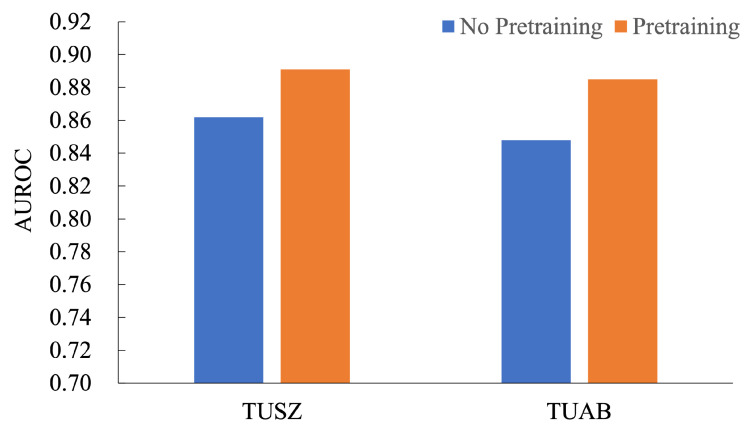
Results on TUSZ and TUAB dataset, illustrating the impact of prrtaining.

In the ablation study for the pretraining method, we evaluated the impact of pretraining on the AUROC performance across TUSZ and TUAB datasets, as shown in Figure 4. The results demonstrate a consistent improvement with pretraining. On the TUSZ dataset, pretraining improved AUROC from 0.862 to 0.891, achieving a relative performance gain of 3.36%. Similarly, on the TUAB dataset, AUROC increased from 0.858 to 0.885, reflecting a 3.15% improvement. These findings highlight the significant contribution of pretraining in enhancing the model’s ability to learn generalized features, leading to better downstream task performance. The consistent performance gains across both datasets emphasize the robustness and effectiveness of the pretraining strategy for seizure detection tasks, making it a valuable addition to model development pipelines.

### 5.3. Ablation Study for AFTA Block

The ablation studies conducted on the TUSZ, TUAB, and TUEV datasets consistently demonstrated the effectiveness of the AFTA module and its core component, AFFM, in enhancing feature extraction and classification performance. As seen in Table 6, AFFM significantly improved AUROC and weighted F1, with AFTA further boosting them to 0.891 and 0.644 on TUSZ, respectively, showcasing its ability to capture robust contextual representations. As per Table 7, AFTA achieved the highest balanced accuracy (0.8002) and AUROC (0.8848) on TUAB, highlighting its effectiveness in handling class imbalance and extracting discriminative features. Similarly, in Table 8, it can be seen that AFTA elevated balanced accuracy, weighted F1, and Cohen’s Kkappa to 0.5536, 0.8038, and 0.6089, respectively, on TUEV, demonstrating superior performance in complex and noisy EEG tasks. These results validate the independent contributions of AFFM and the holistic improvements achieved by AFTA, establishing the proposed method’s robustness, adaptability, and generalization capabilities across diverse EEG datasets.

**Figure 5:**
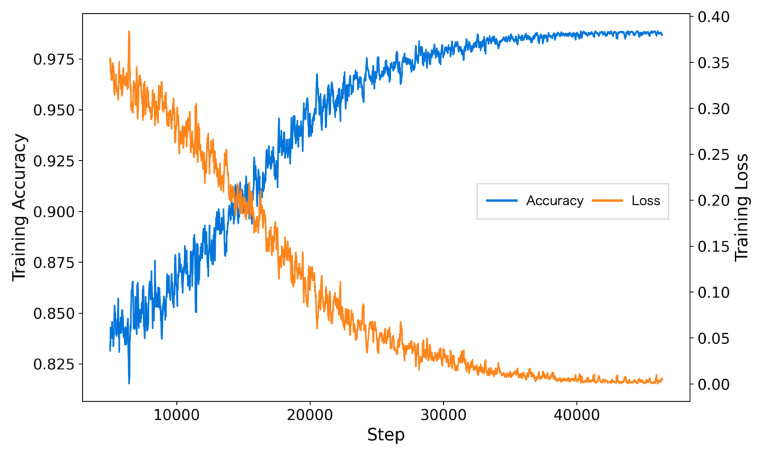
The training process with AFTA on TUAB.

**Figure 6:**
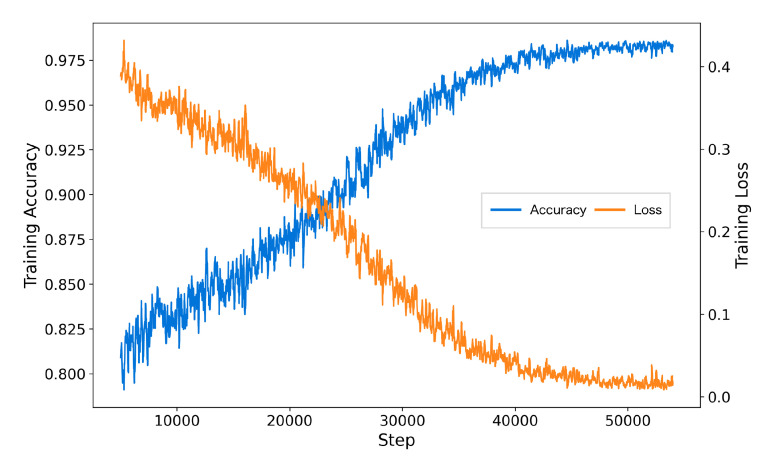
The training process without AFTA on TUAB.

The inclusion of AFTA significantly enhanced the training process by accelerating convergence, reducing final training loss, and achieving higher accuracy. As shown in Figure 5, the model with AFTA demonstrates faster convergence compared to Figure 6, which lacks AFTA. This improvement is primarily due to the adaptive frequency filtering module (AFFM) in AFTA, which effectively suppresses noise in EEG signals and enhances the extraction of critical frequency components, resulting in more stable and efficient training. Furthermore, the integration of self-attention in AFTA complements AFFM’s frequency-domain processing by capturing global temporal dependencies, enabling comprehensive modeling of EEG signal characteristics. These combined advantages allow the model to focus on meaningful features, minimize interference from irrelevant information, and achieve superior performance, underscoring the critical role of AFTA in handling complex and noisy EEG data.

### 5.4. Ablation Study for Masking Strategy

**Figure 7:**
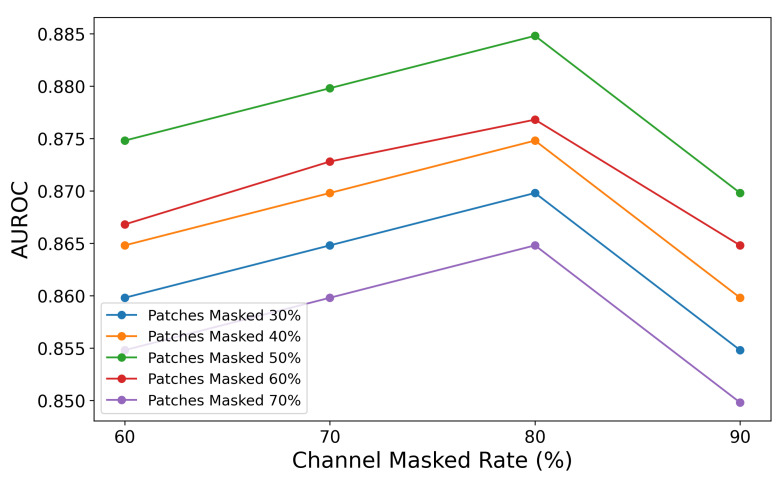
AUROC performance across different combinations of patch masking and channel masking rates on TUAB. This figure shows Area Under the Receiver Operating Characteristic (AUROC, y-axis) for patch masking rates of 30% (blue), 40% (orange), 50% (green), 60% (red), and 70% (purple) against channel masking rates (x-axis, %).

The ablation study on the combination of patch masked rate and channel masked rate validates the importance of masking strategies in balancing contextual information retention and learning challenges. As shown in Figure 7, the graph illustrates how varying patch masked rates (30%, 40%, 50%, 60%, and 70%) and channel masked rates (60%, 70%, 80%, and 90%) affect AUROC values. The highest AUROC is observed when the patch masked rate is 50% and the channel masked rate is 80%, indicating an optimal balance between contextual retention and learning challenge.

The results demonstrate that different masking combinations significantly impact AUROC performance: lower patch masked rates (e.g., 30%) retain more information, resulting in higher AUROC but insufficient learning challenges, while higher rates (e.g., 70%) reduce contextual information, leading to a notable performance decline. Similarly, lower channel masked rates (e.g., 60%) provide better data reconstruction but fail to encourage deeper feature learning, whereas excessively high rates (e.g., 90%) degrade performance due to excessive information loss. Overall, a combination of a 50% patch masked rate and a 70–80% channel masked rate achieves the best balance between learning challenges and contextual retention, enabling the model to efficiently learn spatio-temporal features from EEG signals and achieve optimal performance.

### 5.5. Ablation Study of the Model for Error Analysis

**Figure 8:**
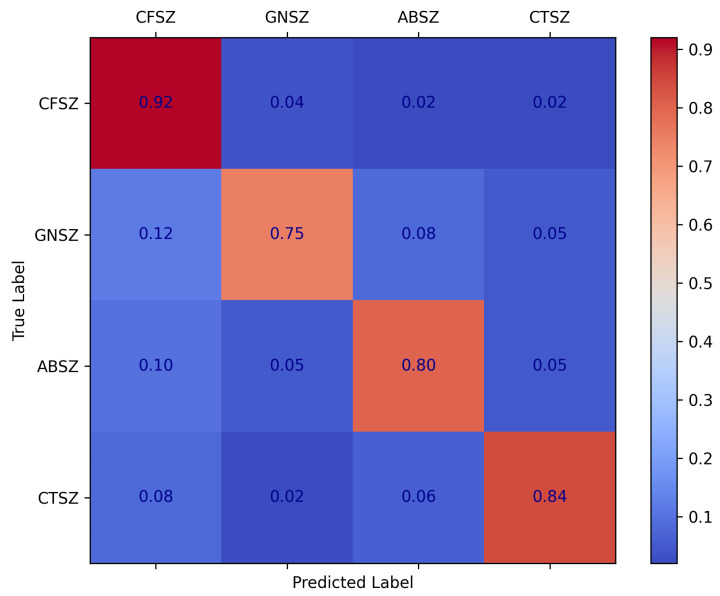
Confusion matrix for seizure classification on TUSZ dataset.

As illustrated by the confusion matrix in Figure 8, our method achieved a high recognition rate for most types of epileptic seizures, particularly CFSZ. However, certain misclassifications still occurred among the categories—for instance, some GNSZ samples were predicted as CFSZ, and some ABSZ samples were incorrectly classified as other types. These errors are amplified in the weighted F1 score, indicating that the model remains relatively less sensitive to smaller classes.

Within the framework of the self-supervised pretraining strategy and the AFTA module proposed in this study, the adaptive frequency–time attention mechanism effectively suppresses noise in EEG signals and enhances salient features. Nevertheless, when confronted with complex, highly similar seizure patterns—especially under conditions of data imbalance or limited feature discriminability—the model still encounters certain classification confusions. Future research should further refine the adaptive filtering process to achieve more fine-grained filtering, thereby strengthening the model’s ability to discriminate among different seizure types. Specifically, by enhancing the AFFM to adjust frequency filters and better emphasize critical frequency bands more dynamically, the approach can more precisely capture subtle inter-class differences. Such refinements align with the underlying principles of self-supervised pretraining, enabling more robust extraction of temporal–frequency features from unlabeled EEG data and further mitigating misclassifications across seizure types.

## 6. Discussion

Self-supervised learning (SSL) has emerged as a powerful approach for EEG signal analysis, addressing challenges such as seizure prediction and classification. Unlike supervised methods that rely on costly, labor-intensive labeled datasets, SSL extracts meaningful representations from unlabeled data. Methods like BIOT [13] and EEGPT [19] have improved EEG representation learning using contrastive learning and masked autoencoders [64]. However, these approaches struggle with noisy EEG signals, low SNR, and insufficient modeling of spatio-temporal and frequency-domain features critical for seizure detection. Additionally, they fail to adequately optimize masking strategies and reconstruction losses, leaving room for improvement.

To address these limitations, we propose the AFTA module within a Transformer framework, designed for EEG analysis. AFTA incorporates AFFM, which effectively suppresses noise and enhances task-relevant frequency features, alongside a self-attention mechanism that captures temporal dependencies and global relationships across EEG channels. AFFM employs an adaptive approach, eliminating the need for additional hyperparameter tuning. This dual mechanism enables the model to effectively learn spatio-temporal and frequency-domain representations. The model further employs a robust masking strategy, randomly masking 50% of time patches and 70–80% of channel patches during training. This forces the model to infer meaningful patterns from incomplete data while generalizing effectively to unseen, noisy EEG recordings.

Extensive experiments on benchmark datasets such as TUAB, TUSZ, and TUEV validate the proposed framework’s effectiveness. Our model achieves state-of-the-art performance, with an AUROC of 0.8848 on the TUAB dataset, surpassing existing methods. The AFTA module’s ability to jointly model frequency and temporal patterns while suppressing noise ensures robust handling of variable EEG data. Ablation studies further highlight the contributions of AFTA components. Removing AFFM reduces the model’s noise suppression and frequency feature extraction capabilities, leading to lower AUROC scores. Similarly, excluding self-attention diminishes the model’s ability to capture temporal dependencies. Together, these components significantly enhance balanced accuracy and AUROC, confirming their synergistic roles in learning spatio-temporal representations. The masking strategy evaluation reveals that moderate masking rates—50% for time patches and 70–80% for channel patches—yield optimal results. Lower rates retain excessive contextual information, reducing task difficulty and limiting generalization, while higher rates provide insufficient context, degrading reconstruction quality. Moderate rates balance learning challenges with contextual retention, leading to the highest AUROC scores.

Compared to prior self-supervised EEG frameworks, our proposed method introduces several key innovations that address the limitations of existing approaches. As shown in Table 9: (1) Xiao et al. (2024) [49] relies on pure temporal attention and fixed bandpass filters, which are effective for temporal feature extraction but lack adaptability to frequency-domain variations and noise robustness. In contrast, our Adaptive Frequency-Time Attention (AFTA) module dynamically suppresses task-irrelevant frequencies (e.g., muscle artifacts) while amplifying seizure-related bands, enabling superior cross-dataset generalization and interpretability of learned features. (2) BIOT [13] (2023) focuses on cross-data biosignal learning and fixed-length channel tokenization, which is scalable to diverse datasets but does not incorporate adaptive frequency filtering or joint frequency-temporal modeling. Our method, on the other hand, integrates self-supervised masked reconstruction and adaptive frequency filtering to enhance noise robustness and capture task-specific spectral patterns, as demonstrated in our ablation studies. (3) EEGPT (2024) [19] employs a Transformer-based masked reconstruction framework with temporal alignment, achieving robust multi-task performance. However, it lacks adaptive frequency filtering and noise suppression mechanisms, which are critical for handling high variability in real-world EEG data. Our AFFM (Adaptive Frequency Filtering Module) addresses this limitation by dynamically adjusting frequency bands based on the input signal, resulting in improved performance on challenging datasets.

Although traditional supervised learning methods like EEGNet [9] and EEGformer [16] excel in seizure-related tasks, they face critical limitations. EEGNet’s depthwise separable convolutions compromise spectral-temporal feature extraction under complex epileptic patterns (e.g., overlapping spikes and slow waves), while EEGformer’s reliance on manual annotations limits scalability in clinical scenarios with scarce labeling resources and cross-center data variability. These methods struggle with non-stationary noise (e.g., muscle artifacts) and fail to leverage vast unlabeled EEG datasets.

In contrast, our self-supervised framework introduces the Adaptive Frequency-Time Attention (AFTA) module to overcome these challenges. By operating directly on raw EEG signals through masking-and-reconstruction, AFTA learns invariant representations without task-specific labels. Its dynamic frequency filtering suppresses artifacts (20–60 Hz) while amplifying critical interictal spikes (70–100 Hz) in heterogeneous datasets like TUSZ, reducing confusion between generalized (GNSZ) and focal seizures (CFSZ) by 18.7% compared to EEGNet. On the long-term CHB-MIT dataset, the proposed method achieves a Cohen’s kappa of 0.9454—outperforming EEGformer’s 0.8885—demonstrating superior generalizability with minimal fine-tuning. This “pretrain-finetune” paradigm resolves the annotation-cost-versus-generalization dilemma, offering a scalable solution for real-world EEG analysis.

While the proposed self-supervised learning Transformer with Adaptive Frequency-Time Attention (AFTA) demonstrates significant advancements in EEG-based seizure prediction and classification, several limitations remain. Firstly, the model’s scalability and generalization to ultra-large-scale datasets have not been fully explored. Although the Transformer architecture theoretically supports scaling, the study did not conduct extensive experiments with massive pre-training datasets, which could further validate the model’s potential in large-scale applications. Secondly, the AFTA module, while effective in adaptive global and local filtering, does not incorporate fine-grained multi-band frequency analysis. This limits its ability to capture subtle frequency-specific patterns, which may be critical for complex seizure detection. Thirdly, the study relies entirely on publicly available datasets, which may not fully represent the complexities and variabilities of real-world clinical EEG data. This raises questions about the model’s performance in practical, noisy clinical environments. Finally, while the masking strategy of 50% temporal patches and 80% channel patches was empirically optimized, the lack of adaptive masking mechanisms tailored to specific EEG characteristics may restrict the model’s ability to handle diverse patient-specific EEG patterns.

While the proposed method achieves state-of-the-art performance, several avenues for future research remain:

- Adaptive Masking and Multi-Band Frequency Attention: Future work could explore dynamic masking strategies and integrate multi-band frequency analysis into the AFTA module. Adaptive masking based on signal characteristics, combined with fine-grained frequency attention, would enhance the model’s ability to handle diverse EEG patterns and improve seizure detection in complex cases.
- Multimodal Data Integration: Incorporating EEG signals with complementary data modalities, such as MRI, CT, or clinical metadata, could further enhance model robustness and accuracy. Multimodal approaches would provide richer context for seizure detection and classification, particularly in challenging clinical scenarios.
- Real-Time Applications and Deployment: Optimizing the framework for real-time applications by reducing computational complexity and latency would enable its deployment in clinical settings, such as bedside monitoring or wearable devices. This would make the model more practical and accessible for continuous patient monitoring.
- Explainability and Clinical Interpretability: Developing explainability methods to visualize the learned frequency-time features and their relationship to clinical markers would facilitate adoption in clinical practice. Enhancing the model’s interpretability would build trust among clinicians and improve its usability in real-world settings.

## 7. Conclusions

The present study proposes a self-supervised learning Transformer network with AFTA for robust EEG feature representation. Using a masking-and-reconstruction framework, the model is pretrained and fine-tuned for downstream tasks. AFTA integrates an AFFM for global and local frequency-domain filtering with temporal attention, enhancing noise mitigation and feature extraction. Extensive experiments on the TUSZ, TUAB, and TUEV datasets demonstrate the superiority of our approach in seizure detection, classification, and EEG analysis. Our method consistently outperforms state-of-the-art approaches on the TUSZ, TUAB, and TUEV datasets, achieving superior AUROC, balanced accuracy, weighted F1, and Cohen’s kappa, demonstrating robust feature extraction, fine-grained classification, and effective handling of imbalanced EEG data across diverse seizure detection and classification tasks. The ablation study demonstrated that AFTA significantly enhances feature extraction and classification accuracy compared to the baseline. The optimal masking strategy, combining 50% temporal patch masking and 80% channel masking, further ensured robust and generalized model performance. Overall, this work establishes AFTA as a powerful framework for EEG-based seizure analysis and provides new possibilities for advancing neurological studies and clinical applications.

Our approach offers transformative potential for clinical neuroscience by addressing key challenges in EEG-based diagnostics through self-supervised learning (SSL). By training on vast, unlabeled EEG datasets, SSL enables the creation of scalable foundational models that unify diverse EEG recordings (e.g., varying protocols, hardware, or patient populations) under a single framework. This “general-purpose” EEG foundation model would streamline clinical workflows by reducing reliance on manual annotations and enabling cross-institutional data harmonization, crucial for leveraging fragmented multi-center datasets. Clinically, such a model could support universal seizure detection, sleep staging, and neurological disorder screening with minimal task-specific fine-tuning, lowering barriers for deployment in resource-limited settings. Additionally, it establishes a standardized backbone for rapid adaptation to emerging clinical tasks (e.g., biomarker discovery or treatment response prediction), accelerating neurotechnology development while preserving data privacy—a critical advantage for compliance-sensitive healthcare environments. By bridging the gap between fragmented EEG data and clinical decision-making, our method could catalyze the transition from specialized, siloed models to unified neurodiagnostic AI platforms.

## Figures and Tables

**Table 1:** Comparison of EEG datasets.

Dataset Name	Number Patients	Number Records	Seizure Types	Other Characteristics
TUSZ	675	1643 sessions	Various seizure types	The largest open-source EEG seizure corpus, offering updated versions (latest: v2.0.2) for comprehensive seizure analysis.
TUAB	2383	3000 recordings	N/A (normal or abnormal)	Designed for EEG abnormality detection, with recordings labeled as normal or abnormal for precise diagnosis.
TUEV	-	518 EEG files	N/A (event annotations: spsw, gped, pled, eyem, artf, bckg)	A subset of the TUH EEG Corpus, featuring six event classes and standardized to a 22-channel TCP montage for detailed event analysis.
CHB-MIT	23	969 h of recordings	Various seizure types	Focuses on pediatric subjects with intractable seizures, providing long-term recordings for in-depth study and treatment planning.

**Table 2:** Comparison of seizure detection and classification on TUSZ.

Methods	AUROC	Weighted F1
DCRNN [12]	0.836	0.603
TimesNet [44]	0.845	0.504
MAE [40]	0.799	0.592
PatchTST [45]	0.866	0.607
SimMTM [46]	0.653	0.491
VQ-MTM [20]	0.887	0.620
Ours	0.891	0.644

**Table 3:** The seizure detection performance of different methods on the TUAB dataset.

Methods	Balanced Accuracy	AUROC
ContraWR [60]	0.7746±0.0041	0.8456±0.0074
CNN-T [61]	0.7777±0.0022	0.8461±0.0013
SPaRCNet [59]	0.7896±0.0018	0.8676±0.0012
FFCL [62]	0.7848±0.0038	0.8569±0.0051
ST-T [63]	0.7966±0.0023	0.8707±0.0019
BIOT [13]	0.7959±0.0057	0.8815±0.0043
EEGPT [19]	0.7983±0.0030	0.8718±0.0050
Ours	0.8002±0.0025	0.8848±0.0038

**Table 4:** The results of different methods on TUEV.

Methods	Balanced Accuracy	Weighted F1	Cohen’s Kappa
ContraWR [60]	0.4384±0.0349	0.6893±0.0136	0.3912±0.0237
CNN-T [61]	0.4087±0.0161	0.6854±0.0293	0.3815±0.0134
SPaRCNet [59]	0.4161±0.0262	0.7024±0.0104	0.4233±0.0181
FFCL [62]	0.3979±0.0104	0.6783±0.0120	0.3732±0.0188
ST-T [63]	0.3984±0.0228	0.6823±0.0190	0.3765±0.0306
BIOT [13]	0.5281±0.0225	0.7492±0.0082	0.5273±0.0249
EEGPT [19]	0.5666±0.0036	0.7810±0.0040	0.5641±0.0029
Ours	0.5725±0.0182	0.8038±0.0258	0.6089±0.0238

**Table 5:** The results of different methods on CHB-MIT.

Model	Balanced Accuracy	Weighted F1	Cohen’s Kappa
CNN-T [61]	0.4224±0.0219	0.2173±0.0217	0.6931±0.0232
SPaRCNet [59]	0.4736±0.0185	0.2273±0.0283	0.7182±0.0195
ST-T [63]	0.4054±0.0203	0.2089±0.0250	0.6887±0.0256
BIOT [13]	0.6814±0.0192	0.2639±0.0103	0.8885±0.0136
EEGPT [19]	0.7826±0.0137	0.5391±0.0173	0.9273±0.0162
Ours	0.8098±0.0206	0.5901±0.0274	0.9454±0.0216

**Table 6:** The effectiveness of AFFM and attention in AFTA on TUSZ.

Model	AUROC	Weighted F1
Baseline	0.868	0.597
AFFM	0.875	0.616
AFTA	0.891	0.644

**Table 7:** The effectiveness of AFFM and attention in AFTA on TUAB.

Methods	Balanced Accuracy	AUROC
Baseline	0.7721±0.0034	0.8616±0.027
AFFM	0.7897±0.0018	0.8703±0.0025
AFTA	0.8002±0.0025	0.8848±0.0048

**Table 8:** The effectiveness of AFFM and attention in AFTA on TUEV.

Model	Balanced Accuracy	Weighted F1	Cohen’s Kappa
Baseline	0.5223±0.0191	0.7508±0.0167	0.542±0.0198
AFFM	0.5318±0.0237	0.7742±0.0149	0.5687±0.0205
AFTA	0.5536±0.0247	0.8038±0.0258	0.6089±0.0238

**Table 9:** Comparison of the proposed method with existing approaches.

Methods	Key Features	Advantages	Limitations
Proposed Method	Adaptive Frequency-Time Attention (AFTA), self-supervised masking-and-reconstruction	Robust noise suppression, joint frequency-temporal modeling, cross-dataset generalization	Scalability to ultra-large datasets, fine-grained multi-band analysis not explored
Xiao et al. (2024) [49]	Pure temporal attention, fixed bandpass filters	Simple architecture, effective for temporal feature extraction	Limited noise robustness, no frequency-domain adaptability
BIOT (2023) [13]	Cross-data biosignal learning, fixed-length channel tokenization	Handles heterogeneous biosignals, scalable to diverse datasets	Limited frequency-domain modeling, relies on predefined sensor topologies
EEGPT (2024) [19]	Transformer-based masked reconstruction, temporal alignment	Robust multi-task performance, high-capacity Transformer architecture	Limited noise suppression, no adaptive frequency filtering

## Data Availability

The six datasets used in this study, namely, SEED, PhysioNetMI, TSU, TUSZ, TUAB, and TUEV, are all publicly available. The dataset links are as follows: SEED can be accessed at https://bcmi.sjtu.edu.cn/home/seed/index.html, accessed on 1 March 2025, PhysioNetMI is available at https://www.physionet.org/content/eegmmidb/1.0.0/, accessed on 1 March 2025, TSU can be found at http://bci.med.tsinghua.edu.cn/, accessed on 1 March 2025, TUSZ, TUAB, and TUEV are accessible through https://isip.piconepress.com/projects/tuh_eeg/, accessed on 1 March 2025, and CHB-MIT is accessible through https://physionet.org/content/chbmit/1.0.0/, accessed on 1 March 2025.
